# An innovation in Flipped Class Room: A teaching model to facilitate synchronous and asynchronous learning during a pandemic

**DOI:** 10.12669/pjms.37.1.3096

**Published:** 2021

**Authors:** Rehana Rehman, Syeda Sadia Fatima

**Affiliations:** 1 Rehana Rehman, PhD. Department of Biological and Biomedical Sciences, Aga Khan University, Karachi, Pakistan; 2 Syeda Sadia Fatima, PhD. Department of Biological and Biomedical Sciences, Aga Khan University, Karachi, Pakistan

**Keywords:** Online learning, Flipped class room, learning experience, in class activity, Student engagement, Virtual learning environment, Synchronous and asynchronous learning

## Abstract

**Objective:**

To conduct an on line Flipped Class Room (FCR) to facilitate synchronous (in class activity) and asynchronous learning [Virtual Learning Environment (VLE)] and acquire feedback of the learning experience from medical students at Aga Khan University.

**Methods:**

This interventional study was conducted with year II students undergoing Endocrine Reproduction Module at the Aga Khan University during April 13 to May 22, 2020. Pre reading material and pretest was shared with the students via Virtual Learning Environment (VLE) one week before the class. Microsoft Teams was used to conduct online session by two facilitators, where student discussion on case studies was encouraged. A WhatsApp group was created with the facilitators to respond to any student queries. The session was recorded and later uploaded on VLE. Posttest and a student satisfaction survey was conducted at the end of the session.

**Results:**

The average score for the pretest and posttest was 19.67 ± 1.37 and 24.60 ± 1.34 respectively (p value <0.05). The learner curve showed an increase in the knowledge learned by 4.93 points (p value <0.05). Fifty-five percent students felt that placement of session was appropriate and were satisfied with the instructions and expected outcomes, received constructive feedback for improvement and generated positive attitude towards learning.

**Conclusions:**

The innovative model of FCR through facilitation of synchronous and asynchronous learning empowered student’s engagement and interactive learning. Students perceived this as a great learning experience which they enjoyed with positive reinforcement from feedback given by the facilitators. They suggested continuation of this model for further learning sessions in other modules of undergraduate medical education at Aga Khan University.

## INTRODUCTION

The COVID-19 pandemic has refocused the world to an era of online learning through use of digital technology and a number of virtual learning platforms (VLP).^1^ Many institutions have nevertheless flipped from traditional to on line teaching to avoid disruption of educational activities. The current situation therefore has created new opportunities for digital learners as well as imposed few challenges for improvement in learning resources, learning designs and faculty’s willingness to embrace technology enhanced learning.^2^,^3^ The requirement of eLearning is growing since students become more and more technologically advanced.^4^

E-learning improves independent learning ability and self-regulating thinking more than the traditional learning modes.^5^ There are two basic types of e-learning; asynchronous and synchronous, with emergent acceptance of synchronous e-learning.^6^ The flipped classroom (FCR) is an active learning pedagogical method that integrates an admixture of asynchronous and interactive synchronous learning strategies. For the asynchronous learning, pre-recorded lectures on learning content, videos, quizzes, and module assignments are uploaded online.^7^ The interactive discussions, and higher-order learning activities like problem-solving then occurs during the class as synchronous component.^8^ FCR has been introduced in different medical universities in different courses suggesting the importance of this approach as appealing method to follow in future years for medical studies.^9^

Considering the challenge of digital teaching and implement a new way of online teaching during the pandemic, we developed FCR model with case-based discussion offering as a live on line session in teams of medical students on Microsoft Teams. We further aimed to assess usefulness of the model in terms of student’s knowledge gain by survey response obtained from the students.

## METHODS

**Fig.1:**
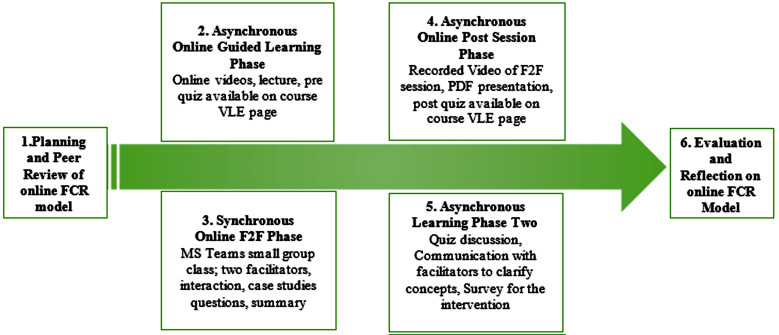
Online FCR flow of events.

This study was conducted at the Aga Khan University with Year II medical students taking the mandatory session in the endocrine reproduction module from April 13 to May 22, 2020. They were informed about the study action plan one week before the session by a class WhatsApp message. The institutional ethics committee approved of the study (ERC# 2019-2048-5368). A written and verbal consent was taken from the students at the start of the session; A brief graphical flow of events is shown in the Fig.1.

### Planning of Session

The two facilitators had six meetings to clarify and finalize the learning outcomes, key concepts, prepare the pre reading material and pre-watching (videos), peer review the teaching plan, modify and revise the clinical scenario. A BOPPPS (Bridge -In, Outcomes, Pretest, Participatory learning, Posttest and Summary) lesson plan was developed and shared for peer feedback and suggestion. Once positive feedback was received, the lesson plan was finalized and submitted to the Academic Year Chair for record. The pre-reading material with the instruction sheet was uploaded on the University’s VLE page specifically designed for the current module.

### Description of Pretest

The pretest comprised of 25 questions, a mixture of C1 and C2 cognition level on all the physiological concepts of “Ovulation, Menstrual Cycle and Implantation” for linking their concepts with the learning objectives of FCR session. A senior faculty member and a clinician was requested to review the tool for its construct and content validity. Items which needed exclusion were highlighted, omissions of repetition was done, double barrel question were removed, discrepancies were remedied, rephrasing of long statements was done to make them simple, clear and unambiguous. A pilot study was conducted, tool was administered to 10 students for its understanding and checking its reliability. Its Cronbach’s alpha was found to be 0.82.

Pretest was closed 48 hours before the session. The facilitators then checked student responses and discussed among themselves the difficult key points (not answered /incorrect answers) by students and used that to ‘bridge in’ their case discussion. A facilitator guide or script with specific key words and time lines was a developed for face to face online session.

### In Class Activity

The class was delivered in two groups via Microsoft Teams. The class was opened by discussing the learning outcomes and the difficult concepts identified during the pretest. Students were divided in teams to respond to facilitator’s questions either by audio or writing in chat box. To generate a debate and discussion amongst the students; sequential disclosure of the patient history, examination and lab investigation merged with key questions was employed by an interactive clinical scenario on “Physiological changes in pregnancy”. Students in each team were given one minute 30 second to respond/counter argue in chat box or over audio. The correct answers were displayed one after the other and required explanation was provided by the facilitator. The total lecture proceedings were recorded and later uploaded on VLE (Virtual Leraning Environment) page for student’s review and revision. A posttest was uploaded on VLE for the students to solve within the next 36 hours to assess the learning curve.

Student’s response on usefulness was acquired by a questionnaire on a 7-point Likert Scale 0-6 (uncommented, sometimes, to some extent, fairly often, very frequently, almost always, and always) uploaded on VLE. No response was received for the ‘uncommented’ score. Once all students had completed the tasks both pre and post tests were made available to students with answer key and feedback. In addition, students were able to discuss difficulties and clarify concepts over WhatsApp with the facilitators.

Data was analyzed using the SPSS statistical package, version 21.0. (IBM Corp. Armonk, NY). Means with standard deviation were calculated or pre and post-test scores. Paired sample t-test was applied on the scores of pre- and post-tests to compare the effect of online intervention. The feedback received by student was reported as frequencies and percentages. A P-value of less than 0.05 was considered as significant.

## RESULTS

The detialed results are shown in Tables-I-II. Total number of students attending the session were nintey eight. The average score for the pretest was 19.67 ± 1.37 points while for posttest was 24.60 ± 1.34 points. The learner curve shows an increase in the knowledge learned by 4.93 points (p<0.05) (Table-I). Most questions in both pre and post tests were positive discriminators with exception of Q2, Q6, Q7 and Q 10 (pre and posttest respectively). Table-II A-D shows the survey results. Sum of student responses who marked in the category of almost always and always: 56 students felt that the placement of session was appropriate and; 64 felt that enough preparation time was provided, 72 and 57 were satisfied with the instructions and the expected outcomes. Similarly, students who scored 5 or higher for application of knowledge; 63 students felt that the activity was able to link content effectively, 56 and 58 said it helped them retain the new gained knowledge respectively, and 54 felt that it stimulated learning. For self-accountability, students who scored 5 or higher 47 felt motivated, 45 came prepared for the session, 59 felt accountable for their own learning, 43 dedicated time for the preparatory activity and 42 felt accountable for the team work.

**Table-I:** Details of the Pre and Post Test.

	Pre test	Post Test
Number of complete graded first attempts	101	101
Absolute Score	19.67 ± 1.37	24.60 ± 1.34
Total Number or Questions	25	27
Difference/Gain of score between Pre and Post test Score	4.93 (p<0.05)	

Data presented as Mean and Standard Deviation. Comparison between scores was done by paired T test. A P value of <0.05 was considered significant.

**Table-II:** Survey Results.

I. Non-face to face Component n= 98						

	Sometimes	To some extent	Fairly often	Very frequently	Almost always	Always
Placement in schedule was appropriate	8 (8%)	2 (2%)	9 (9%)	24 (24%)	32 (33%)	23 (23%)
Time allocated was adequate	2 (2%)	1 (1%)	9 (9%)	22 (22%)	31 (32%)	33 (34%)
Schedule given well before time	0	1 (1%)	7 (7%)	18 (18%)	33 (34%)	39 (40%)
Expected outcomes outlined	1 (1%)	7 (7%)	10 (10%)	23 (23%)	30 (31%)	27 (28%)
Working groups pre-defined	0	2 (2%)	9 (9%)	26 (27%)	28 (29%)	33 (34%)

**II. Application Exercises n= 98**						

Applied linking of concepts	2 (2%)	1 (1%)	15 (15%)	17 (17%)	32 (33%)	31 (32%)
Aided retention of concepts	1 (1%)	3 (3%)	15 (15%)	23 (23%)	27 (28%)	29 (30%)
Assisted knowledge sharing	1 (1%)	6 (6%)	15 (15%)	18 (18%)	29 (30%)	29 (30%)
Stimulated problem-solving skills	1 (1%)	5 (5%)	16 (16%)	22 (22%)	25 (26%)	29 (30%)

**III. Self Accountibility n= 98**						

Motivated to come prepared	5 (5%)	5 (5%)	20 (20%)	21 (21%)	26 (27%)	21 (21%)
Came fully prepared for the class	4 (4%)	4 (4%)	13 (13%)	33 (34%)	27 (28%)	17 (17%)
Felt accountability for learning	1 (1%)	5 (5%)	16 (16%)	17 (17%)	36 (37%)	23 (23%)
Dedicated more time in preparation	2 (2%)	9 (9%)	17 (17%)	27 (28%)	20 (20%)	23 (23%)
Exhibited accountability in team work	6 (6%)	8 (8%)	20 (20%)	22 (22%)	25 (26%)	17 (17%)
**IV. Student satisfaction n= 98**						
Enjoyed learning activities	2 (2%)	4 (4%)	14 (14%)	19 (19%)	34 (35%)	25 (26%)
Enhanced student’s engagement	4 (4%)	8 (8%)	12 (12%)	21 (21%)	28 (29%)	25 (26%)
Received constructive criticism	4 (4%)	10 (10%)	12 (12%)	26 (27%)	21 (21%)	25 (26%)
Developed positive attitude	2 (2%)	8 (8%)	10 (10%)	24 (24%)	28 (29%)	26 (27%)
Interested to participate in similar activities	4 (4%)	8 (8%)	7 (7%)	17 (17%)	34 (35%)	28 (29%)

Data presented as absolute values with percentages in parenthesis.

Lastly, we inquired about student satisfaction; students who scored 5 or higher 59 students enjoyed the session, 53 were engaged throughout, 47 received constructive feedback for improvement, 54 were able to generate positive attitude towards learning and 62 felt that similar activities should be conducted for other concepts.

## DISCUSSION

The importance of on-line learning has been highlighted by a number of studies across the medical education continuum.^10^,^11^ However it is blamed for being of lower quality, resource intensive and technical expertise of teaching and learning staff required in comparison to learning by face-to-face interaction.^12^ COVID-19 pandemic has provided us an opportunity to design VLP which has now been used effectively by students as well as teachers.^13^ The execution of task requires a paradigm shift, with focus on available resources, selection of teaching methodologies, mentoring of faculty, communication with the concerned IT personnel, troubleshooting and correction after receiving feedback.

In this scenario, we planned our teaching model by consecutive meetings of faculty with the IT personnel and academic year chair to design a model for the technology savvy medical students.^14^ A VLP was used to provide instructions, send video links and test their knowledge by a pretest. The preparedness of students was established from responses of pretest. The decorum of ‘in class’ was maintained by guide lines / ground rules for online interaction, and step by step instructions provided by facilitators.^11^ Students liked the ‘Bridge-In Activity’ which clarified miss-concepts and constructed knowledge on the incorrect responses obtained by pretest.

We made clinical case scenarios on physiological changes in pregnancy, presented with sequential events and encouraged students to take part in interactive discussion through chat box. The selection of clinical cases was on the basis of their importance for learning of concepts through guided enquiry and closeness to the practical life.^5^,^11^ The interactive session during the sequential discussion of clinical case supported students engagement which is concomitant to positive learning outcomes of critical thinking and affirmative grades.^15^,^16^ This active interaction in an online discussion forum is a key to support effective teaching and learning.^17^ Students were satisfied and mentioned that the activity enabled self-directed learning with construction of knowledge and rectification of mistakes an interactive format as has been mentioned in the literature.^15^,^18^ Students were encouraged to provide effective feedback on this model on WhatsApp, VLE and email which gave a chance to teachers to review, clear and explain the miss concepts.^19^ The facilitators responded to their queries on any clarification concepts in posttest. Second thing was that the online in class activity session was recorded was uploaded on the VLE page to substantiate asynchronous learning. Students prefer to revisit video/summary or lecture at their leisure time and this fact/point was also echoed by our participants as highlighted by the student comment “*It is easy for us to go back and go through the whole video for a summary or even revising it*” This fact is also well documented in literature.^11^ The national studies reiterate the use of online learning in medical and dental institutes, with improvement of learning during this pandemic situation.^11^ However students have requested necessary measures for improving e-teaching for better learning during this period 20. Keeping in mind these perspectives our study will be a stepping stone to document experiences of on line learning in FCR.

### Limitations of the study

Medical education literature on interventional studies support that improvement in learning can be facilitated with any educational intervention that needs comparison while planning the respective study. Our study is limited by lack of comparison with any other teaching /learning methodology. Furthermore, limitations of the study were sample population derived from a single university, only year II MBBS students and only one topic of the module which defered generalizability of results. Nevertheless, we have added an innovation to FCR, a temptation for both online and offline learning and a combination of VLE all efforts directed to improve student satisfaction and learning.^19^,^20^

## CONCLUSION

In the middle of fears, myths, threats and challenges of COVID-19, we developed an on-line FCR model with discussions on clinical cases in teams of medical students that facilitated synchronous as well as synchronous learning through in class activity and VLP. Students expressed satisfaction in terms of knowledge construction through the pre-rereading material, in class activity and guidance received from facilitators after the post test. The in-class activity was particularly appreciated by students who enjoyed the learning experience and suggested this for being implemented in further learning sessions.

### Authors Contribution:

**RR and SSF:** Conceived, designed and did statistical analysis & editing of manuscript.

**RR and SSF:** Did data collection and manuscript writing.

**RR and SSF:** Did review and final approval of manuscript. Both authors attest to the accuracy or integrity of the work published.
